# Global inequities and political borders challenge nature conservation under climate change

**DOI:** 10.1073/pnas.2011204118

**Published:** 2021-02-08

**Authors:** Mark A. Titley, Stuart H. M. Butchart, Victoria R. Jones, Mark J. Whittingham, Stephen G. Willis

**Affiliations:** ^a^Department of Biosciences, Durham University, DH1 3LE Durham, United Kingdom;; ^b^BirdLife International, CB2 3QZ Cambridge, United Kingdom;; ^c^Department of Zoology, University of Cambridge, CB2 3EJ Cambridge, United Kingdom;; ^d^School of Natural and Environmental Sciences, Newcastle University, NE1 7RU Newcastle, United Kingdom

**Keywords:** climate change, biodiversity, transboundary, conservation, political borders

## Abstract

Underlying sociopolitical factors have emerged as important determinants of wildlife population trends and the effectiveness of conservation action. Despite mounting research into the impacts of climate change on nature, there has been little consideration of the human context in which these impacts occur, particularly at the global scale. We investigate this in two ways. First, by modeling the climatic niches of terrestrial mammals and birds globally, we show that projected species loss under climate change is greatest in countries with weaker governance and lower Gross Domestic Product, with loss of mammal species projected to be greater in countries with lower CO_2_ emissions. Therefore, climate change impacts on species may be disproportionately significant in countries with lower capacity for effective conservation and lower greenhouse gas emissions, raising important questions of international justice. Second, we consider the redistribution of species in the context of political boundaries since the global importance of transboundary conservation under climate change is poorly understood. Under a high-emissions scenario, we find that 35% of mammals and 29% of birds are projected to have over half of their 2070 climatic niche in countries in which they are not currently found. We map these transboundary range shifts globally, identifying borders across which international coordination might most benefit conservation and where physical border barriers, such as walls and fences, may be an overlooked obstacle to climate adaptation. Our work highlights the importance of sociopolitical context and the utility of a supranational perspective for 21st century nature conservation.

## Significance

Ecological communities are undergoing a major redistribution as species track their moving climatic niches on a warming planet. This has major repercussions for global biodiversity governance. By simulating the changing distributions of species under climate change, and comparing impacts between nations, we highlight the global inequities in climate impacts on nature. We then consider the global importance of transboundary conservation under climate change, as many species ranges are projected to move across political borders. By mapping transboundary range shifts globally, we highlight regions where international cooperation may be most useful for conservation and where border barriers may be most detrimental. Our findings underscore the need for cooperation across national boundaries to minimize biodiversity loss in the face of global change.

Earth’s biodiversity is set to face major disruption under climate change, with substantial implications for natural ecosystems and human societies that depend on them (1–4). However, the fate of biodiversity depends not only on the severity and distribution of climate impacts, but also on the human context in which they occur (5). For example, socioeconomic factors such as governance, corruption, and conflict frequency are important predictors of wildlife population trends and the effectiveness of conservation efforts (6–9). Political borders, too, have important conservation implications where they fragment policy and legislation across species ranges (10) or where they present physical barriers to movement (11–14). Here, we use ensemble niche modeling to investigate climate-induced biodiversity change in the context of these two key human considerations: socioeconomic factors of relevance for biodiversity conservation and the political borders that circumscribe and delineate their influence.

## National Sociopolitical Context of Climate Impacts

**Fig. 1.**
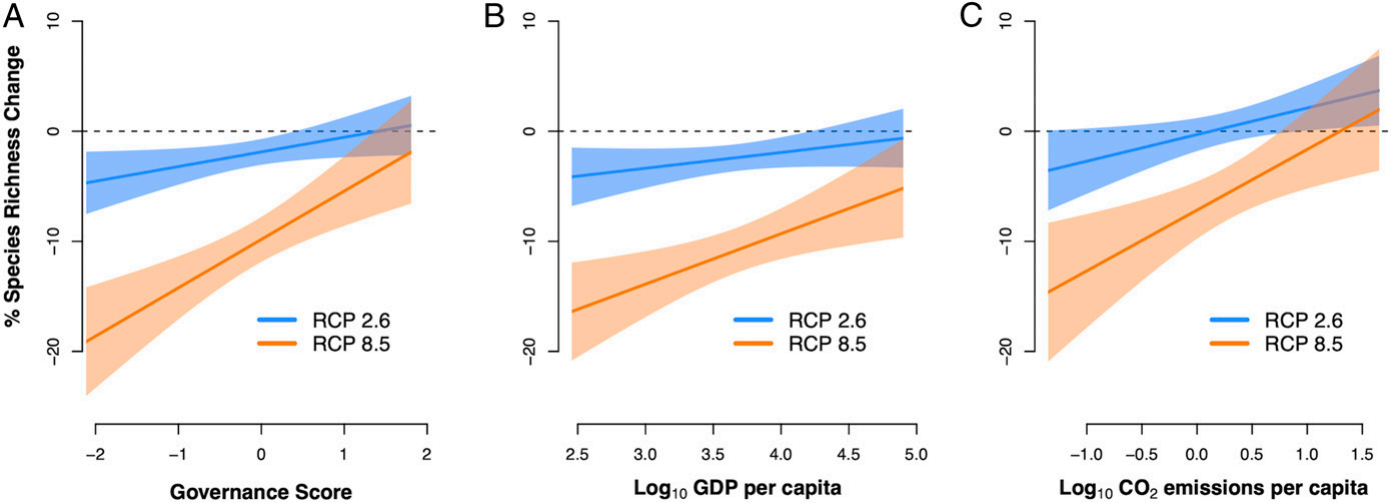
National context of projected climate impacts on birds and mammals. Modeled relationships between mean percentage change in species richness for each country (projected to 2070) and national-level socioeconomic datasets: (*A*) governance score, (*B*) per capita GDP, and (*C*) per capita CO_2_ emissions. In each case, results are shown for a low (RCP 2.6) and a high (RCP 8.5) emissions scenario; see *SI Appendix*, Fig. S1, for two intermediate scenarios (RCP 4.5 and RCP 6.0). For *A* and *B*, relationships shown are for birds and mammals combined, while *C* shows the relationship for mammals only (since the percentage species richness change for birds was not significantly related to CO_2_ emissions in either RCP scenario). For full GLM results, coefficient estimates, and significance values, refer to *SI Appendix*, Table S1. Shaded areas illustrate 95% confidence bands. Governance score is the mean value of the six national-level worldwide governance indicators provided by the World Bank for the year 2018, which are standardized scores that range from −2.5 to 2.5, where a lower score indicates weaker governance, and which capture government effectiveness; control of corruption; political stability and absence of violence; rule of law; regulatory quality; and voice and accountability (15).

We modeled the climatic niches of >12,700 species (around 80%) of terrestrial mammals and birds—two groups whose distributions are well-characterized—excluding species with highly restricted ranges whose distributions are likely determined by factors other than climate (*Materials and Methods*). We projected species climatic niches to 2070 under the four emissions scenarios adopted by the Intergovernmental Panel on Climate Change (IPCC) for the Coupled Model Intercomparison Project (CMIP5) (Representative Concentration Pathway [RCP] 2.6, RCP4.5, RCP6.0, and RCP8.5) (2). For our first strand of analysis, we aggregated our projections from a half-degree resolution to the national level and related the projected changes in species richness to national level data on governance, per-capita Gross Domestic Product (GDP), and CO_2_ emissions. As an indicator of governance, we used a score derived from six World Bank governance indicators (15) which has been shown to predict conservation success globally (7). This score reflects survey respondent views on dimensions of governance such as control of corruption, government effectiveness, and political stability (15). Under medium (RCP 4.5 and RCP 6.0) and high (RCP 8.5) emissions scenarios, relative loss of bird and mammal richness is greater in countries with lower governance scores and lower per-capita GDP (Fig. 1 and see *SI Appendix*, Fig. S1, for RCP 4.5 and RCP 6.0). Therefore, birds and mammals may be most threatened by climate change in the countries currently with the potentially lowest capacity to implement effective conservation. We also found that loss of mammal species is projected to be greater in countries with lower per-capita CO_2_ emissions—the countries least responsible for climate change in the first place (Fig. 1 and *SI Appendix*, Fig. S1).

These patterns reflect a tendency toward greater impacts from climate change in low-latitude countries, which also tend to rank lower for governance, GDP, and CO_2_ emissions. Although the magnitude of climatic changes is projected to be greatest at higher latitudes (2), climate impacts on nature may be greater in tropical areas because these areas are more likely to see the emergence of novel climates (16) and are also where species have narrower climatic niches, making them more sensitive to change (17). These global inequities in climate impacts on nature reignite questions surrounding the morality of climate inaction in developed nations, which have benefitted disproportionately from fossil fuel consumption—and which continue to benefit from global biodiversity conservation—but face fewer of the associated impacts and costs (18). Our results further strengthen the case for substantial and urgent climate change mitigation action, which would minimize these inequities in climate impacts on nature (Fig. 1).

## Conserving Birds and Mammals across Political Borders

In our second strand of analysis, we examined the distributions of birds and mammals in relation to political borders, considering both their present distributions and their projected distributions under climate change. Political borders demarcate the spatial extent of territory ownership and governance, and, by extension, influence the distribution of threats to biodiversity (10). Consequently, populations of the same species occurring on either side of a political border can be exposed to different threats and pressures, with different implications for conservation and management. Borders can also reduce the efficiency and effectiveness of conservation by impeding coordinated conservation action on either side, especially in areas of conflict (10, 19). These concerns, combined with an increasing appreciation for the broad scale at which ecological processes operate, have led to the growth of the “transboundary conservation” paradigm in recent decades (11). For example, the Convention on the Conservation of Migratory Species of Wild Animals (CMS), or the Bonn Convention, was established to coordinate international conservation strategies across the ranges of migratory species (20). Under climate change, however, the ranges of many nonmigratory species are likely to shift across international borders, too, requiring supranational conservation strategies that are similarly coordinated between nations for perhaps a much larger suite of species (21–23).

**Fig. 2.**
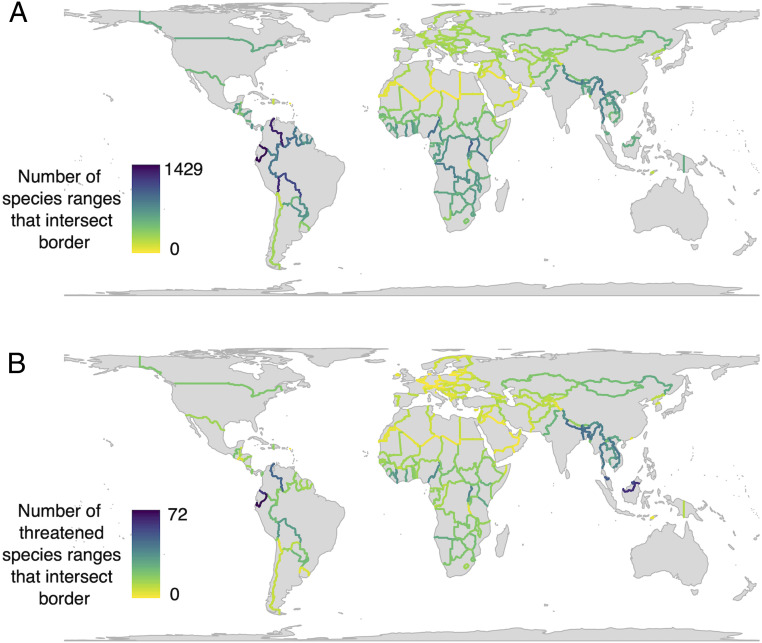
Global transboundary species richness. Maps of the number of species (*A*) and threatened species (*B*) whose ranges intersect with political borders. Darker borders indicate a greater number of species that have their ranges bisected by that border.

Despite this growing impetus for internationally coordinated conservation, there has been limited effort to characterize the importance of transboundary conservation globally (24) or to identify places where it would be most beneficial in the context of climate change. To address this gap, we first intersected the current distribution maps of all of the world’s terrestrial mammals and birds with maps of the world’s political borders to calculate the number of species ranges that each border currently bisects. This highlights borders across which transboundary conservation would benefit the most species, such as in the western Amazon and central Africa (Fig. 2*A*). We repeated this for threatened species to reveal borders where cross-border conservation effort might be prioritized (Fig. 2*B*). By dividing transboundary richness by the total number of species found in the countries on either side of each boundary, we also highlight regions where a disproportionate number of species ranges intersect political borders (*SI Appendix*, Fig. S2). This emphasizes areas such as western and southern Africa and central Europe, where a high proportion of the species found there span multiple countries. We also calculated that the majority of mammals (60.03%) and birds (71.63%) are “transboundary” in the sense that their ranges span multiple countries and cross international boundaries, underscoring the importance of cross-border collaboration if conservation is to succeed in reversing biodiversity declines.

**Fig. 3.**
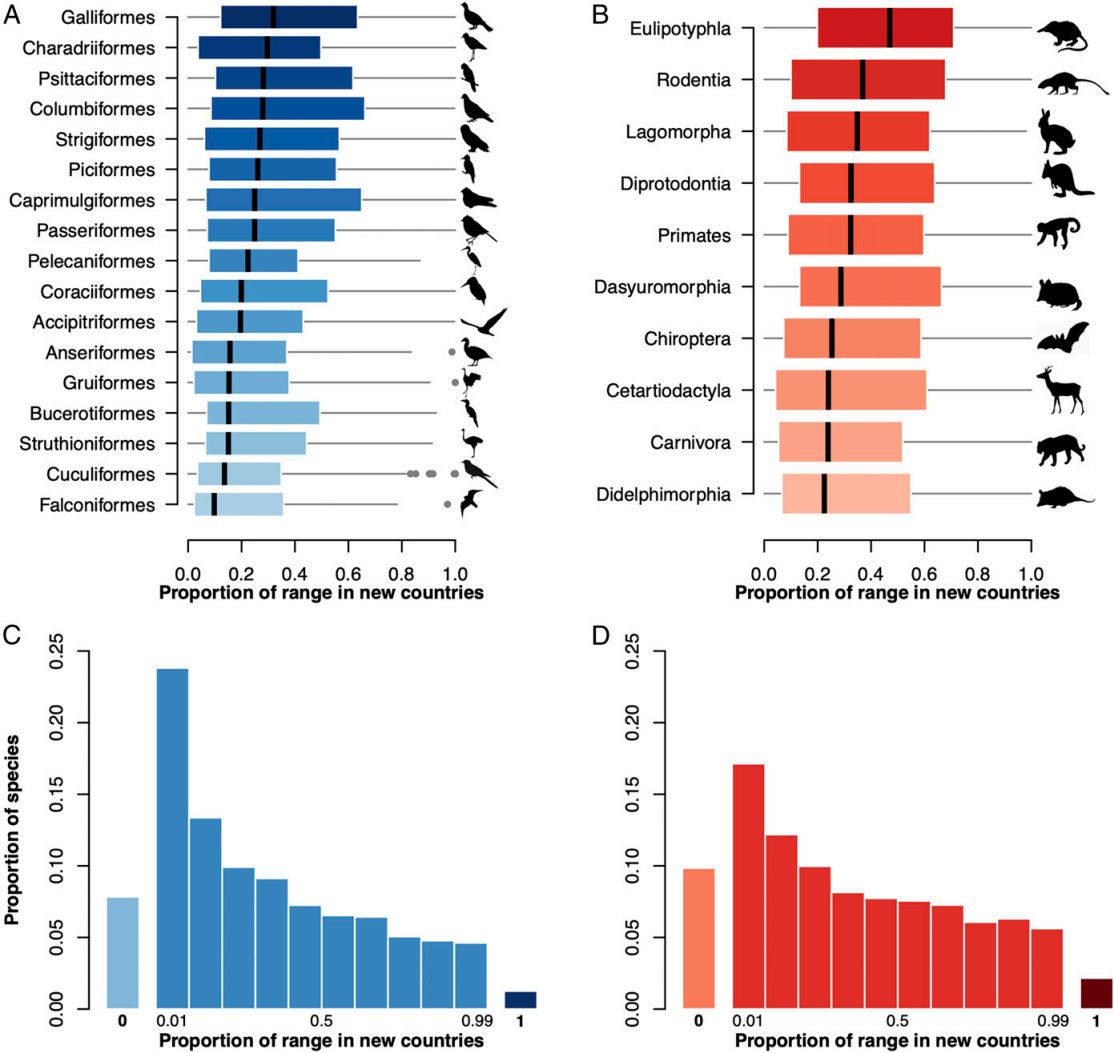
Proportion of species whose ranges move into “new” countries. Boxplots illustrate the proportion of species ranges that are projected to be found in “new” countries (countries in which the species is not currently known to occur) under a high emissions scenario (RCP 8.5) in 2070. Results are broken down by taxonomic order for birds (*A*) and mammals (*B*). For clarity, only orders with 50 or more modeled species are shown. (*C* and *D*) Histograms show the proportion of all modeled birds (*C*) and mammals (*D*) with a given proportion of their 2070 range in new countries under RCP 8.5. Bars are plotted separately (labeled 0 and 1) for the special cases in which species are projected to have none or all of their future niche in new countries. See *SI Appendix*, Fig. S3, for the equivalent results under a lower-emissions scenario of RCP 4.5.

Climate change increases the importance of transboundary conservation efforts because many species ranges may shift across political borders to track their climatic niche, with important implications for international biodiversity governance (21–23). In tracking their climatic niche into new countries, species may be afforded more or less effective conservation across their range owing to differences in conservation policy between countries (22, 25). To explore this possibility, we combined our projections of species climatic niches with spatial data on the world’s political borders. This revealed that, under a high emissions scenario (RCP 8.5), our models project 35.0% (1343) of mammals and 28.7% (2559) of birds to have more than half of their future (2070) climatic niche in countries in which they are not currently found (Fig. 3). Furthermore, over half of modeled mammals (60.8%, or 2,336) and birds (55.0%, or 4,904) have at least one-fifth of their future climatic niche in such “new” countries (Fig. 3 and see *SI Appendix*, Fig. S3, for moderate emissions scenario RCP 4.5).

**Fig. 4.**
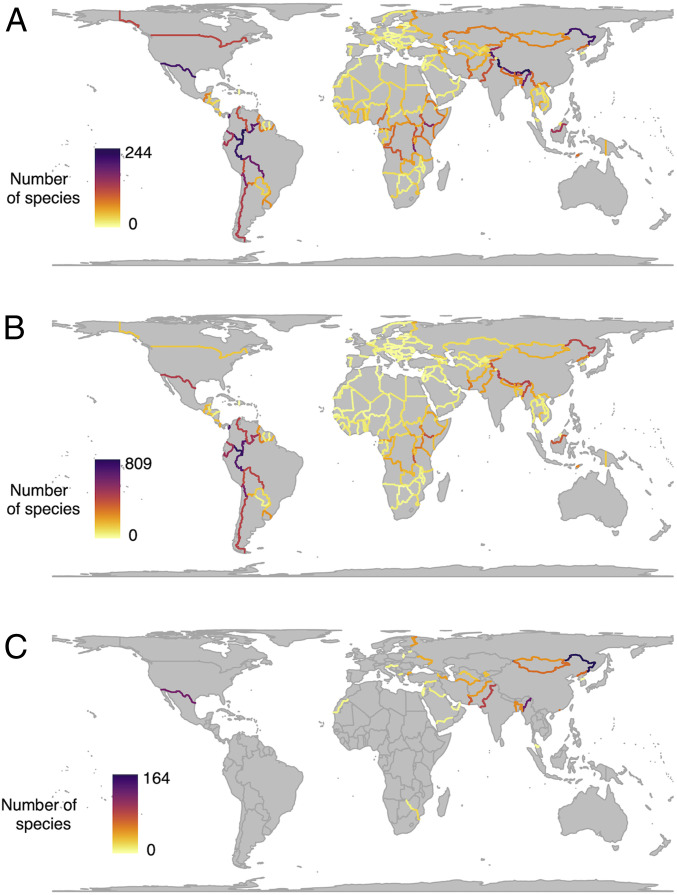
Projected transboundary range shifts for terrestrial mammals and birds under climate change (RCP 8.5). Maps of the world’s political borders colored according to the number of (*A*) mammals and (*B*) birds whose ranges are projected to cross that border by 2070 (in either direction). For mammals, transboundary range movement is highest in western Amazonia, the United States–Mexico border, central and east Africa, the China–Russia border, and the Himalayan region. For birds, western Amazonia is the focus of transboundary range shifts. For a moderate emissions scenario of RCP 4.5, see *SI Appendix*, Fig. S3. (*C*) Political borders with barriers (complete or under construction) along them, colored according to the number of nonflying mammal species whose ranges are projected to cross that border by 2070 (in either direction).

By summing the number of species whose climatic niches move into adjacent countries for each political border, we were able to map transboundary range shifts globally (Fig. 4 and *SI Appendix*, Fig. S4). For mammals, key regions where species may move into new countries under climate change are the United States–Mexico border, western Amazonia, the Andes, central and eastern Africa, the Himalayan region, and the China–Russia border. For birds, western Amazonia emerges as the focus of transboundary range movement. Our results highlight how species-rich regions with political borders that cut across latitudinal or altitudinal climatic gradients are likely to be hotspots for transboundary range shifts and suggest that, under climate change, this is where proactive cooperation on nature conservation will be most beneficial. In some regions, particularly where governance and cross-border collaboration are already weak and human pressures are high, this will be challenging. The projections are perhaps therefore more troubling for mammals, given the higher numbers of projected transboundary shifts in regions recently identified as having low feasibility for transboundary conservation (24), such as central and eastern Africa, parts of the Middle East, and borders around the Bay of Bengal. We also repeated this analysis controlling for species richness (by dividing the number of transboundary shifts by the total species richness across the two countries involved). This highlighted areas with high transboundary movement relative to their species richness, for example, the Argentina–Chile border, eastern Africa, and the Middle East (*SI Appendix*, Fig. S5). Together, our results indicate that transboundary conservation efforts, while already important for many species, will be of increasing value under climate change. Bilateral or multilateral cooperation will be needed, particularly across borders that we project to be crossed by many range shifts (Fig. 4) or where range shifts are projected to be particularly large (*SI Appendix*, Fig. S6*A*). As priorities in these areas, we suggest preserving or augmenting habitat connectivity across such borders; expanding and updating the identification of Key Biodiversity Areas for species (26); coordinating transboundary-protected area network design and management to account for the needs of range-shifting species; coordinating appropriate legislation (such as hunting controls for targeted species); expanding the identification and monitoring of range-shifting species; and using or creating means of sharing knowledge, resources, and skills between nations.

Transboundary range shifts are likely to have socioeconomic and management implications for the countries involved (3, 27). Range shifts of key “charismatic” species, for example, could make countries more or less appealing for wildlife tourism, with economic consequences for countries that rely on wildlife tourism as a significant source of income. Our models suggest that transboundary range shifts of such species are most likely in central Africa, the western Amazon, and the Himalayas (*SI Appendix*, Fig. S6*B*). For species threatened by wildlife trade, such as those listed under the Convention on International Trade in Endangered Species of Wild Fauna and Flora, transboundary range shifts may be especially important, since they may move into countries that offer more or less protection by domestic legislation. This may particularly affect species in the Americas (*SI Appendix*, Fig. S6*C*).

The prospect of significant species redistributions has led to calls for new multilateral conservation treaties to meet the demands of biodiversity governance in the 21st century. However, establishing bilateral and multilateral transboundary conservation initiatives takes considerable time and financial resources and is likely to be particularly challenging where current national-level conservation capacity is weak or lacking (21, 24, 28). Fortunately, many structures are already in place to coordinate conservation policy between multiple governments. The CMS, for example, sets out the legal basis for coordinating conservation strategies across migratory species ranges. Agreements or less formal instruments within the CMS require signatories to take into account the need for collaborative transboundary measures with adjacent states (29, 30); to cooperate regionally and internationally to remove barriers to migration (31, 32); to identify transboundary habitats that could be considered “transfrontier conservation areas” (32, 33); and to ensure physical and ecological connectivity between sites now and under climate change (31, 34). Building upon or making innovative use of existing mechanisms such as these to consider explicitly species whose ranges shift across political borders under climate change, for a broader suite of species, may enable more rapid progress. Furthermore, strengthening coordination across borders need not be restricted to top–down legislative action. Particularly in regions where international relations are poor, or where national-level governance and conservation capacity are weak, less formal and locally established approaches may be more successful at enhancing cooperation and protecting species and habitats that span international borders (24, 35, 36).

## Border Barriers and Conservation under Climate Change

Political borders present a more tangible conservation challenge where they are fortified with a physical barrier, such as a wall or fence. As of 2012, 13.2% (by length) of the world’s borders are marked with a physical barrier of some form, totaling over 32,000 km (37), and the past two decades have seen a surge in the planning and construction of fortified political borders (38). However, the ecological implications of these barriers have not been investigated on a global scale. The construction of such barriers can disturb or destroy habitats, fragment populations, prevent dispersal and migration, and directly or indirectly increase mortality via entanglement, poaching, and predation (10, 12–14, 39, 40). For example, border security fencing in Central Asia is likely to be impeding ungulate migrations (11, 41), while recently erected razor-wire security fencing along the Slovenia–Croatia border has increased mortality in herons and ungulates (42). Barriers along stretches of the United States–Mexico border were found to decrease the abundance of puma (*Puma concolor*) and coati (*Nasua narica*) (43), and the planned extension of this barrier is likely to prevent the re-establishment of dwindling or recently extirpated populations of endangered species in the United States, such as the Mexican gray wolf (*Canis lupus baileyi*) and Sonoran pronghorn (*Antilocapra americana sonoriensis*) (12). Under climate change, border barriers may present an additional threat if they prevent species from tracking and filling their shifting climatic niche, but to our knowledge this possibility remains unexplored.

To explore the global ecological implications of border barriers, now and under climate change, we compiled a list of border barriers around the world that are built or are under construction (*SI Appendix*, Fig. S7). By intersecting these fortified borders with species distribution data, we calculated that they intersect the ranges of, and so may be an obstruction to dispersal for, 775 species (18.5%) of nonflying mammals. The United States–Mexico border wall alone would bisect the ranges of 120 nonflying mammals. While fortified borders similarly intersect the ranges of 264 species of bats and 2,337 species of birds, we assume that most are capable of dispersing over border barriers, but note that some terrestrial and understorey forest specialist bird species have very low dispersal ability across roads, rivers, and other linear clearings (44, 45). A radiotelemetry study of ferruginous pygmy owls (*Glaucidium brasilianum*) near the United States–Mexico border, for example, revealed that their reluctance to fly far above the ground would cause a border wall combined with vegetation gaps to obstruct transboundary movement (39). Furthermore, although our analysis has focused on mammals and birds, the implications of political borders and border barriers for nature conservation extend to other taxonomic groups, too. Amphibians and reptiles may be negatively affected, while low-flying insect species may be affected by less permeable structures such as walls (12).

Considering projected range shifts under climate change, under RCP 8.5, our models show that 696 species (16.24%) of nonflying mammals may be unable to track their climatic niche into new countries because of existing (or under-construction) border barriers. These are species whose current climatic niche is found on one side of a fenced or fortified border, and their 2070 climatic niche is projected to cross it. The United States–Mexico border barrier, as a noteworthy example, would prevent 122 species from tracking their climatic niche into the adjacent country. The potential ecological impacts on regional biodiversity that the United States–Mexico border wall may inflict have been previously highlighted (12, 39). However, our analysis suggests that its impacts could be more damaging still under climate change and that, from this ecological standpoint, it may be one of the worst international borders on the planet along which to build such a wall (Fig. 4 and *SI Appendix*, Fig. S8). Along with the United States–Mexico border, two other fortified borders are of particular ecological concern: the India–Myanmar border fence, which is under construction, and the China–Russia border. These three border barriers rank at the top for the number of species whose climatic niches are projected to cross them (Fig. 4*C*) and remain at the top of the list when the proportion of species ranges that cross them are taken into account (*SI Appendix*, Fig. S8). In the case of the United States–Mexico and China–Russia borders, this is likely to be because these are long east-west–oriented barriers that could intercept latitudinal range shifts under climate change. The India–Myanmar border would likely impact many species due to its position perpendicular to an elevation gradient in an important biodiversity hotspot (46). We also examined which groups of species may be most affected by border barriers by breaking down the results by taxonomic order (*SI Appendix*, Fig. S9). This revealed carnivorans, ungulates, and lagomorphs to have the highest proportion of species whose ranges are projected to cross a border barrier—a third or more of modeled species in these orders. This is concerning given that these groups are known to be among the most vulnerable to border-fencing impacts (14). To mitigate these impacts, barriers should be made as permeable to wildlife as possible, enabling smaller animals to pass though or underneath, and openings should be strategically placed to allow larger animals to cross between countries (11, 47). When and where necessary, assisted translocation of species across borders could be considered to help facilitate range shifts under climate change. Ecologists must participate in the debates surrounding border fortification to ensure that the full costs and benefits of this infrastructure can be taken into account.

## Conclusions

We highlight three broad insights from our analysis combining macroecological modeling with global sociopolitical considerations. First, climate impacts on biodiversity are skewed toward the countries with potentially lower capacity for effective conservation and less culpability for climate change in the first place. This is morally and practically important for global biodiversity governance given that similar inequities in the causes and impacts of climate change have been a key obstacle in multilateral climate negotiations (48). Second, the pervasiveness and magnitude of projected transboundary shifts among bird and mammal species mean that safeguarding Earth’s biodiversity under climate change will demand much greater cross-border collaboration from local communities, conservation organizations, and national governments than is needed at present. To facilitate this at the supranational level, expanding or drawing lessons from existing multilateral mechanisms such as CMS, where international cooperation is already a central tenet, may be a pragmatic way forward. Finally, maintaining and enhancing habitat connectivity across borders between area-based conservation measures will be critical to enable range shifts under climate change, and this effort should be targeted to the regions in which it will have the most impact. We have shown that this is likely to be where borders cut across broad climatic gradients in species-rich areas, such as around the tropical Andes and Amazon, the Himalayas, and east–west borders such as the United States–Mexico border. Where border security barriers are a threat to this ecological connectivity, particularly along the United States–Mexico border and parts of Asia, we must ensure that appropriate measures are in place to mitigate their impacts. As climate change drives the displacement of both wild species and humans (2, 49), barriers intended to constrain the movement of people must not have unintended adverse outcomes for the natural world.

## Materials and Methods

### Modeling Climatic Niches.

Our approach focused on ensemble species distribution modeling. Also known as bioclimatic envelope or niche modeling, this method depends on statistical associations between species distributions and environmental variables. Projected changes in environmental variables (due to climate change, for example) can then be used to infer changes in the distributions of species climatic niches. When used appropriately, the approach has been shown to accurately simulate responses to climate change for mobile species (50–52).

### Species Distribution Data.

Species distribution data were obtained from the International Union for Conservation of Nature (IUCN) Red List (53) for 5,381 species of terrestrial mammals and from BirdLife International and *Handbook of the Birds of the* World (54) for 10,930 species of birds. The range polygons were filtered to keep only “Extant” or “Probably Extant” polygons (“Presence” code 1 or 2) where the species was native (“Origin” code 1) for the species breeding and resident ranges (“Seasonality” code 1 or 2). The resulting range polygons were then rasterized to a grid with 0.5° resolution. Grid cells were classed as Presence where they had at least 10% overlap with the range polygon. To avoid the inherent problems when modeling the climatic niches of range-restricted species (where climate is less likely to be an important determinant of the species distribution), we excluded species classified as being present in fewer than 10 grid cells. This resulted in a final set of 3,840 mammal and 8,918 bird species—78.2% of the original species. All modeling was done in a cylindrical equal area projection to avoid biasing the models by oversampling high latitudes (55). For each species, 1,000 pseudoabsence points were randomly sampled from the same zoogeographic realm(s) (56) in which the species was found. Points were sampled from the same zoogeographic realm to minimize sampling from regions that are climatically suitable but where the species is not found because of geographical barriers such as oceans and large mountain ranges. We chose a relatively coarse scale (0.5°) to model species climatic niches because climate is ecologically relevant for species distributions at broader scales and because climatologists often caution about the accuracy of climate data derived from General Circulation Models at finer spatial scales (57). Moreover, at this scale we can be reasonably confident that range margins are broadly accurate for species, even in less-well-recorded regions.

### Predictor Variables.

Despite the significant body of research employing species distribution models, the bioclimatic predictor variables used vary widely, with little consensus on the best approach to select them. One common approach is to use all 19 bioclimatic variables provided by the Worldclim dataset (58, 59), although high intercorrelations between the variables can lead to model instability (and issues with assigning causality) and are particularly problematic when projecting to future climate scenarios and/or different geographic regions (60). A preferable approach is to select variables that are ecologically relevant to the species being modeled based on expert knowledge of causal relationships (61, 62). However, this option was unfeasible on a global scale, since there is no obvious set of predictors of ecological relevance to all species, and a lack of species-specific knowledge prevents the identification of relevant variables for every species individually. Consequently, we use a systematic approach to select a set of predictor variables that are broadly ecologically relevant and noncollinear and that produce high-performing models when tested on a random subset of species.

First, we preselected eight bioclimatic variables from the WorldClim dataset (59) that have been widely used in niche modeling and have been used to model species distributions accurately under a changing climate (e.g., ref. 51). These included mean annual temperature and precipitation, temperature seasonality, precipitation seasonality, maximum temperature of the warmest month, minimum temperature of the coldest month, precipitation of the wettest month, and precipitation of the driest month. For both temperature and precipitation, these eight variables capture the annual typical conditions, variability (seasonality), and extremes. We then generated all possible combinations of these eight variables, in combinations of between three to eight variables. This resulted in 219 possible combinations (1 combination of 8, 8 combinations of 7, 28 combinations of 6, and so on). Of these 219, 10 sets were discarded because they did not contain both temperature and precipitation variables. The remaining 209 sets of variables were then tested for collinearity; if any variables in the set had pairwise correlations of *r* > 0.7 (60), the set was discarded. This left a final selection of 38 candidate combinations of predictor variables that are biologically relevant at a coarse scale and sufficiently uncorrelated to avoid producing unstable models. These 38 combinations were then used to build Generalized Additive Models (GAMs), using the R package mcgv (63), for a random subset of 200 bird and 200 mammal species (for more detail, see GAM modeling methods below in Model Validation). The 38 candidate combinations were then ranked according to model performance (using Akaike Information Criterion) to identify the best set of predictor variables by tallying the number of times that set appeared in the top quartile of candidate sets. The final set included the following five predictors: mean annual temperature, temperature seasonality, precipitation of the wettest month, precipitation of the driest month, and precipitation seasonality. This set was in the highest performing quartile of candidate variable combinations for >90% of mammal and bird species. When projecting future climate variables, we used downscaled General Circulation Model (GCM) data from CMIP5, downloaded from WorldClim (59). To take into account variation in climate projections between different climate models, we used outputs from three different GCMs (HadGEM2-ES, CCSM4, and MIROC-ESM-CHEM).

### Spatial Autocorrelation.

Spatial autocorrelation (the higher similarity of closer samples) is a pervasive phenomenon in ecological data. If present and unaccounted for in model development, spatial autocorrelation can lead to inaccurate estimation of model coefficients, inflation of significance values, and inappropriate spatial inference and prediction (64–66). To account for the spatial dependence in our models, we split the gridded presence/absence data for each species into 10 spatially disaggregated blocks (67). Noncontiguous portions of the world’s terrestrial ecoregions were used as the sampling units to divide the data; these units were then grouped into 10 blocks using the blockTools package in R (68) such that the total area and mean bioclimate was approximately equal in each block and that each block contained the full range of bioclimates (67).

### Model Validation.

By splitting the data into 10 blocks, we were able to use 10-fold cross-validation to assess model performance. Each block was left out in turn to be used as a testing dataset, and models were trained on the remaining 90% of data. Model performance was then assessed using the area under the curve (AUC) of the receiver operator characteristic curve, which tests for discrimination ability.

### Ensemble Climatic Niche Models.

Here, our focus is not on projecting realized distribution changes but rather on exploring the potential for species climatic niches to shift into novel regions. For this purpose, we considered that simple species distribution models are adequate. The potential limitations of species-distribution models in projecting actual range shifts for species are widely recognized and have been comprehensively discussed elsewhere (62, 69, 70). Future model development could incorporate species traits, land use, and biotic interactions. However, currently we lack sufficient data for almost all species in relation to limiting biotic interactions and their roles in determining species realized niches. Similarly, global projections of future land use and land cover are currently available only at very coarse spatial scales and for such broad habitat classifications, so as to be inappropriate for inclusion in modeling future scenarios for most species.

Adapting the methods of Bagchi et al. (67), we used an ensemble of four different model types for each species: generalized linear models (GLMs), GAMs, random forests, and boosted regression trees (BRTs). Combining an ensemble of models has been demonstrated to reduce overfitting and improve predictive performance, especially for rare species (71, 72). The four model types were selected to provide contrast between regression-based and machine-learning techniques. This methodology has previously been shown to model species distributions accurately (51, 67, 73). Models were fitted on training data leaving one block out in turn, resulting in 40 models per species (10 blocks × 4 model types). These models were then used to project future climatic niches across the same and adjacent zoogeographic realms (56) using future climate variables from the three selected GCMs. This resulted in 120 projections per species (40 models × 3 GCMs) for each emissions scenario. Projected probability of occurrence was converted into a binary presence–absence value using a threshold that maximized sensitivity plus specificity (74). The final projected distribution was determined by taking the mean presence–absence value for each grid cell, weighted by AUC, to give greater influence to better-performing models in the ensemble. The models had good discrimination ability, with mean AUC (± SD) of 0.942 (± 0.052) for mammals and of 0.941 (± 0.049) for birds. Details of model formulation for each model type are given in more detail below; all modeling was done using R (75).

#### Generalized linear models.

When fitting GLMs, we optimized the combination of polynomial model terms to maximize model performance in terms of AUC for each species, as follows. GLMs were used to fit up to and including third-order polynomials for the five predictor variables, resulting in 243 candidate model formulations. Models were fitted to nine blocks of data, with the remaining block used as a testing dataset to evaluate AUC. This was then repeated for each of the 10 data blocks. The combination of polynomial terms that maximized AUC across the 10 model fittings was used to fit a final set of 10 models.

#### Generalized additive models.

We used thin-plate regression splines to fit GAMs using the mgcv package in R (63). These regressions were fitted as a Bernoulli response using a logit link function. Once again, models were fitted on 90% of the data, leaving one block out as a testing dataset to assess model performance using AUC.

#### Random forests.

Random forest modeling was done using the package randomForest in R (76). The number of variables (“mtry”) randomly sampled at each split was allowed to vary between one and three. The number of trees was then set initially to 1,000, and a random forest was fitted to the data, sequentially omitting one block of data for cross-validation so that performance could be assessed using AUC. The number of trees was then increased by 500, and the procedure was repeated until the increased performance (from the addition of 500 new trees) measured using AUC was <1%. The values of mtry and the number of trees that maximized model performance (averaged across the 10 blocks of omitted data) were used to fit the final set of 10 models.

#### Boosted regression trees.

Boosted regression trees were generated using the gbm R package (77). A similar cross-validation approach was used to parameterize the BRT models. Learning rate (also known as the shrinkage parameter) was set at 0.001; the number of trees was set at 5,000; and tree complexity was allowed to vary between 1 and 4. The tree complexity that minimized summed error across the testing data blocks was used to fit a final set of 10 models.

### National-Level Socioeconomic and Emissions Data.

To investigate the sociopolitical context of our projections, we first generated grid-cell-level projections of species richness under the current climate and the 2070 climate for each RCP scenario by summing for each grid cell the number of species for which it contains a suitable climate. We then calculated the projected percentage change (the present to 2070) in species richness for each grid cell. To aggregate these grid-cell-level projections at the national level, we took the mean across all grid cells in a country. We then related this measure of national-level richness change to three socioeconomic datasets of relevance to wildlife conservation and climate change: governance, per capita GDP, and per capita CO_2_ emissions.

To generate a national level measure of governance quality, we used the six Worldwide Governance Indicator datasets provided by the World Bank (15). These included indicators of Political Stability and Absence of Violence, Control of Corruption, Government Effectiveness, Rule of Law, Regulatory Quality, and Voice and Accountability. These governance indicators are based on a range of underlying data and are aggregate scores that combine the views of enterprise, citizen, and expert survey respondents; for full methodology, see ref. 78. Since these six measures of governance are highly correlated with one another (see *SI Appendix*, Fig. S10 for a Principal Components Analysis of the six variables), we took the mean across all six to produce a single national-level governance metric. This aggregate score has previously been shown to be the strongest predictor of population declines and conservation success in waterbird populations globally (7).

For all variables, 2018 data were used as the most recent complete dataset, with the exception of CO_2_ emissions data, for which 2014 data were the most recent data available. To model global patterns of biodiversity change with these socioeconomic factors, we used generalized linear models in R. See *SI Appendix*, Table S1, for detail of GLM formulations and parameters.

### Political Borders Analysis.

We obtained spatial data on the world’s political borders using the R package rworldmap (75, 79). To calculate the number of species ranges that intersect with the world’s political borders, we intersected species-range polygons with the political borders dataset using the raster R package (80). To map projected transboundary niche movement, we identified borders across which “new” species may appear from adjacent countries because their climatic niche is projected to cross the border. To identify political borders that have physical barriers along their length, we first used those listed in refs. 11, 37 and updated the list with an internet search to identify those added since the date of publication (2012). We also included barriers currently under construction or proposed, since they have the potential to affect biodiversity in the time period of our modeling (the present to 2070).

## Supplementary Material

Supplementary File

## Data Availability

Study data to reproduce the analysis are accessible online from the Environmental Information Data Centre (DOI: 10.5285/5bf972a8-c9a3-4721-8089-552dfe3ff124) (81). Species distribution data are available from the IUCN Red List and BirdLife International and the *Handbook of the Birds of the World* (53, 54). Bioclimatic data, including future projections, are available to download from WorldClim (59) (http://wordclim.org/data/index.html). World Bank governance indicators (15) are available to download at https://info.worldbank.org/governance/wgi/.
