# Supplementary material for: Global inequities and political borders challenge nature conservation under climate change
Source: Proc Natl Acad Sci U S A. 2021 Feb 8;118(7):e2011204118. doi: 10.1073/pnas.2011204118 (PMC7896304; doi:10.1073/pnas.2011204118)
Supplement: Supplementary File [file pnas.2011204118.sapp.pdf]

# Supporting Information for

Global inequities and political borders challenge nature conservation under climate change

**Authors**

Mark A. Titley, Stuart H. M. Butchart, Victoria R. Jones, Mark J. Whittingham, Stephen G. Willis

**Corresponding authors**

* Mark A. Titley and Stephen G. Willis

**Email:**

mark.a.titley@durham.ac.uk

s.g.willis@durham.ac.uk

**This PDF file includes:**

Figures S1-S11
Table S1

**Supporting Figures and Tables**

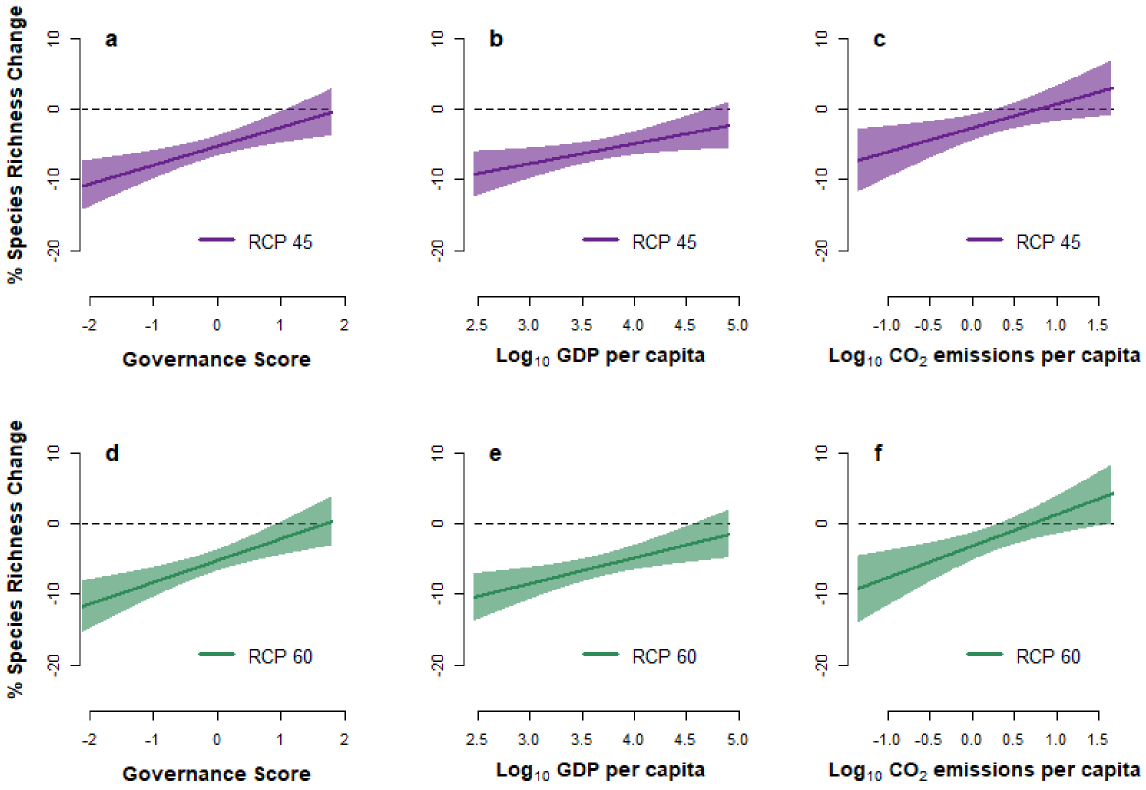


**Figure S1. National context of projected changes to terrestrial mammal and bird richness for RCP 4.5 and RCP 6.0.** Lines show modelled linear relationships between mean percentage change in species richness across all grid cells in each country and the country's governance score (a and d), GDP (b and e), and $CO_2$ emissions (c and f). a-c are under RCP 4.5 and d-f under RCP 6.0. As with Fig 1, relationships for bird richness change with $CO_2$ emissions were not statistically significant (see Table S1) so panels c and f show relationships with mammal richness change only. All others are for combined mammal and bird richness change. Shaded areas indicate 95% confidence bands.

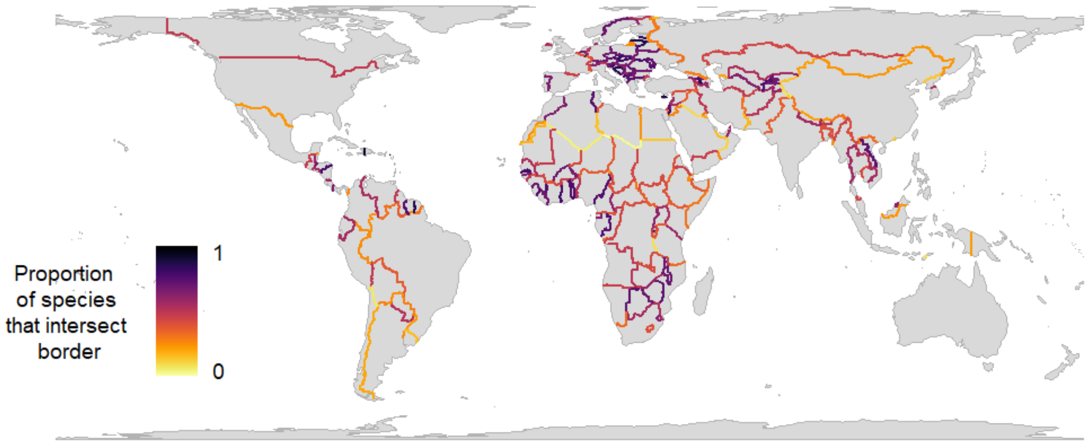


**Figure S2. Relative transboundary species richness.** Borders are coloured according to the proportion of species found in the two countries either side of the border whose ranges intersect the border. This highlights areas where many of the species are 'transboundary', such as western and southern Africa and central Europe. In such areas, transboundary conservation efforts may benefit a disproportionate number of species.

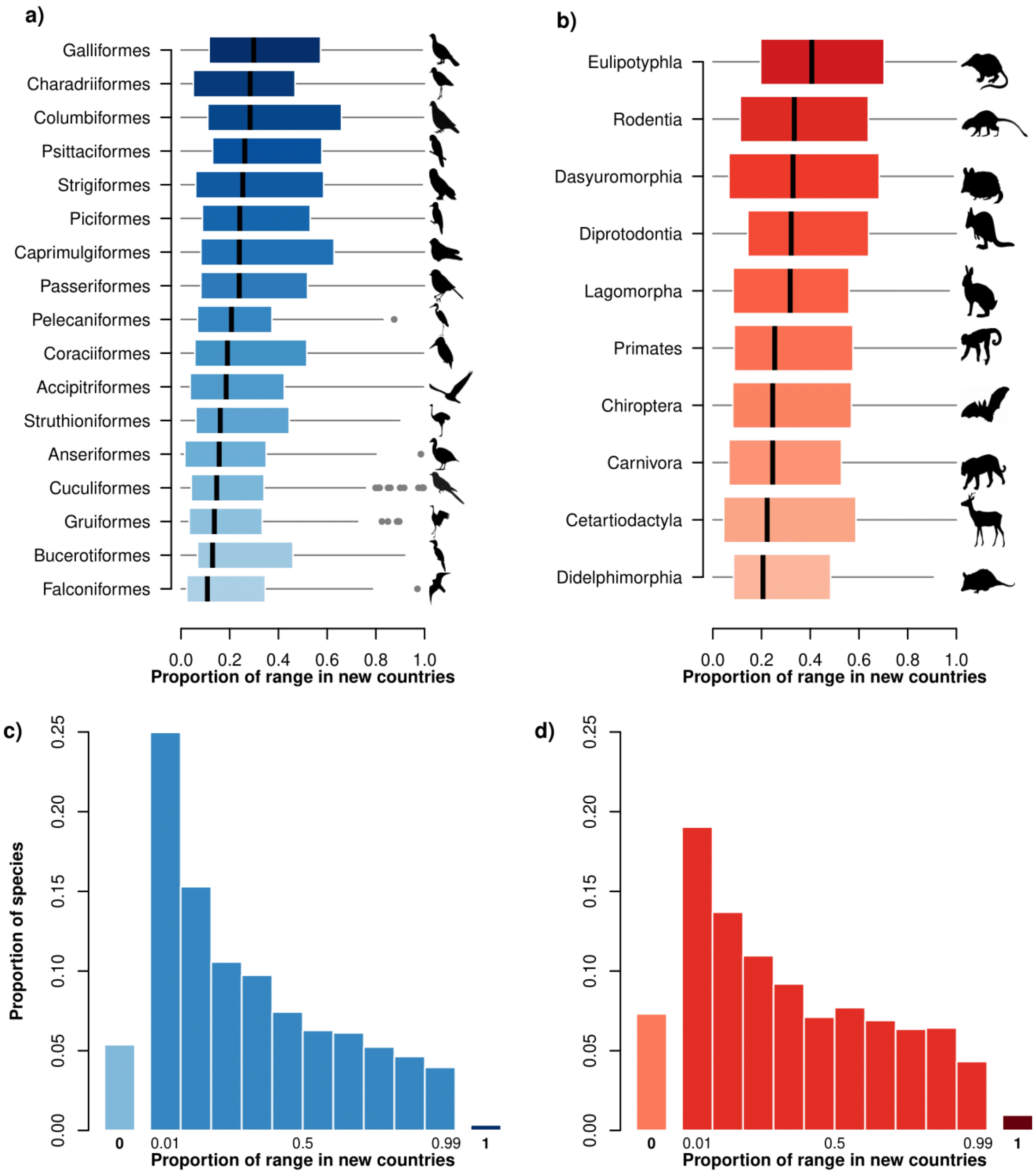


**Figure S3: Proportion of species ranges found in 'new' countries in 2070 under a moderate emissions scenario (RCP 4.5)**. Boxplots show the proportion of species ranges in 'new' countries (countries in which the species is not currently found) for birds (a) and mammals (b) broken down by taxonomic order. For clarity, only orders with 50 or more modelled species are shown. Beneath, histograms show the proportion of all modelled birds (c) and mammals (d) with a given proportion of their 2070 range in 'new' countries, under RCP 4.5. Bars are plotted separately (labelled 0 and 1) for the special cases in which species are projected to have none or all of their future niche in new countries

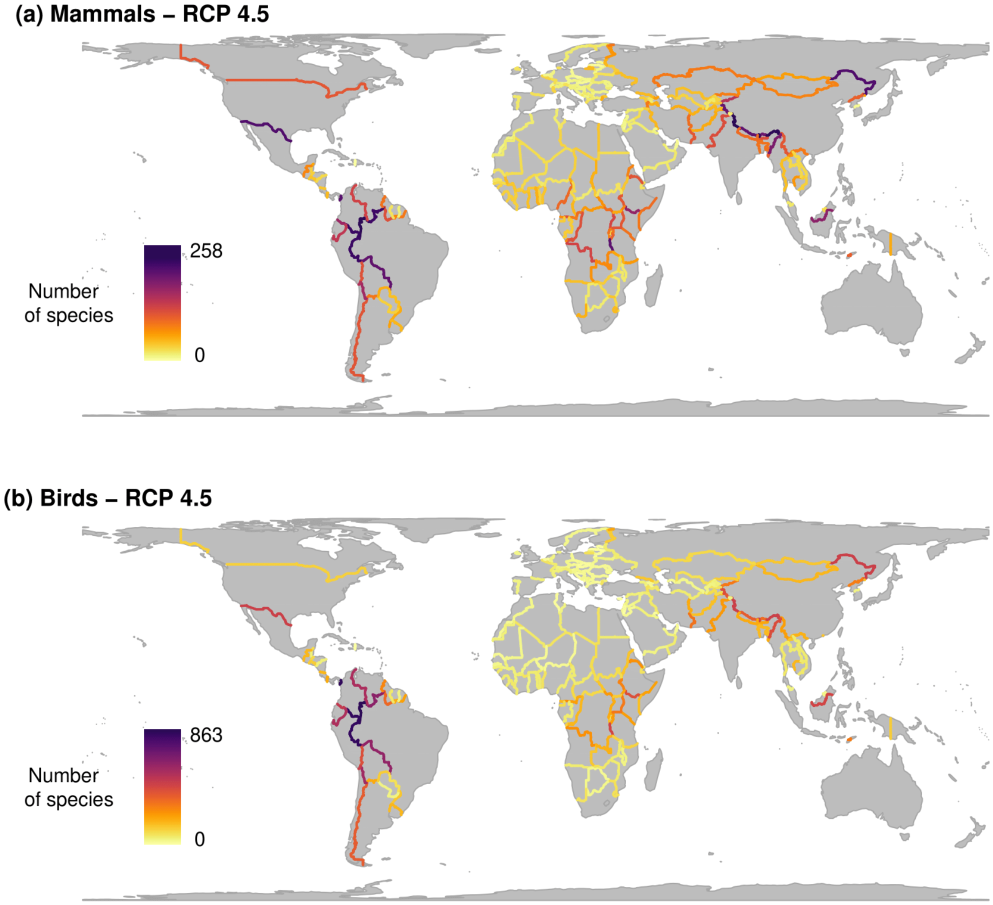


**Figure S4. Projected transboundary range shifts for terrestrial mammals and birds under climate change under a moderate emissions scenario (RCP 4.5).** National political borders are coloured according to the number of mammal or bird species whose ranges are projected to cross that border under RCP 4.5.

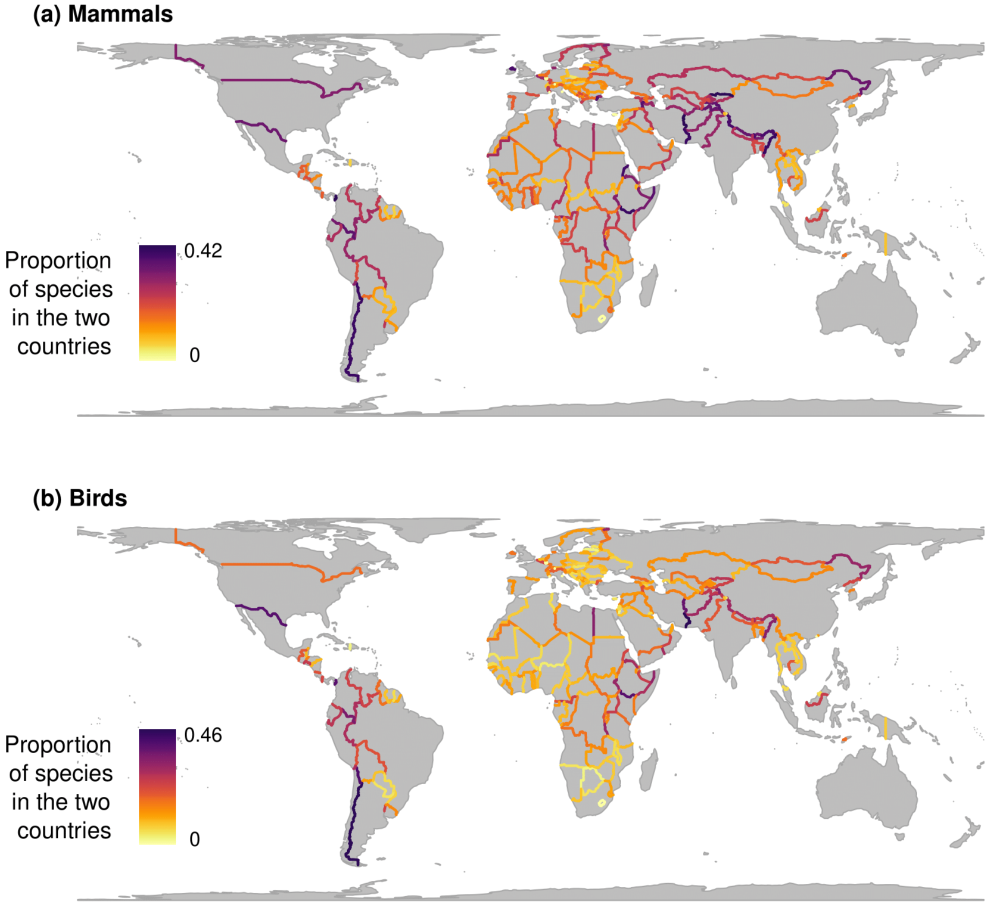


**Figure S5. Transboundary range shifts relative to species richness.** Borders are coloured according to the number of species' of (a) mammal and (b) bird whose ranges are projected to move across each political border under RCP 8.5, relative to the total number of species of bird or mammal in each country pair.

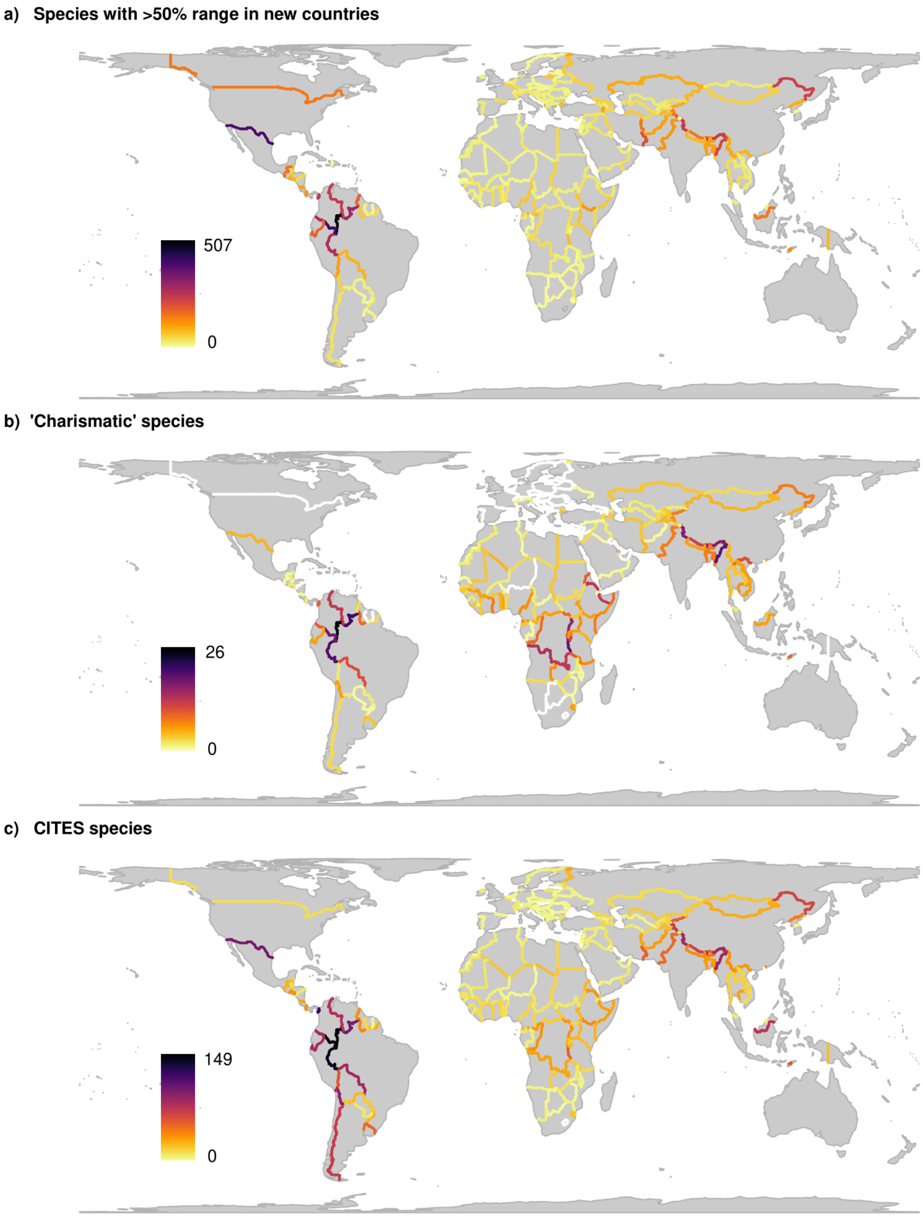


**Figure S6: Transboundary range shifts for selected subsets of species.** Projected transboundary range shifts (2070, RCP 8.5) are shown for three subgroups of species of particular conservation interest: species that have the majority of their range in new countries (a), ‘charismatic’ groups of mammals species that are likely to be economically valuable for wildlife tourism (b), and species listed under the appendices of CITES (Convention on International Trade in Endangered Species) (c). The ‘charismatic’ species considered in (b) include mammals Felidae (cats), Ursidae (bears), Hyaenidae (hyeanas), Rhinocerotidae (rhinoceroses), Elephantidae (elephants), and Canidae of the genera *Canis* and *Lycaon* (wolves and painted dogs), along with the common hippopotamus, giraffe, African buffalo and the three zebra species (82–84). We also included primates from the families Hominidae (great apes), Cebidae (capuchins and squirrel monkeys), Atelidae (howler, spider and woolly monkeys), Cercopithecidae (Old World monkeys) and Hylobatidae (gibbons)

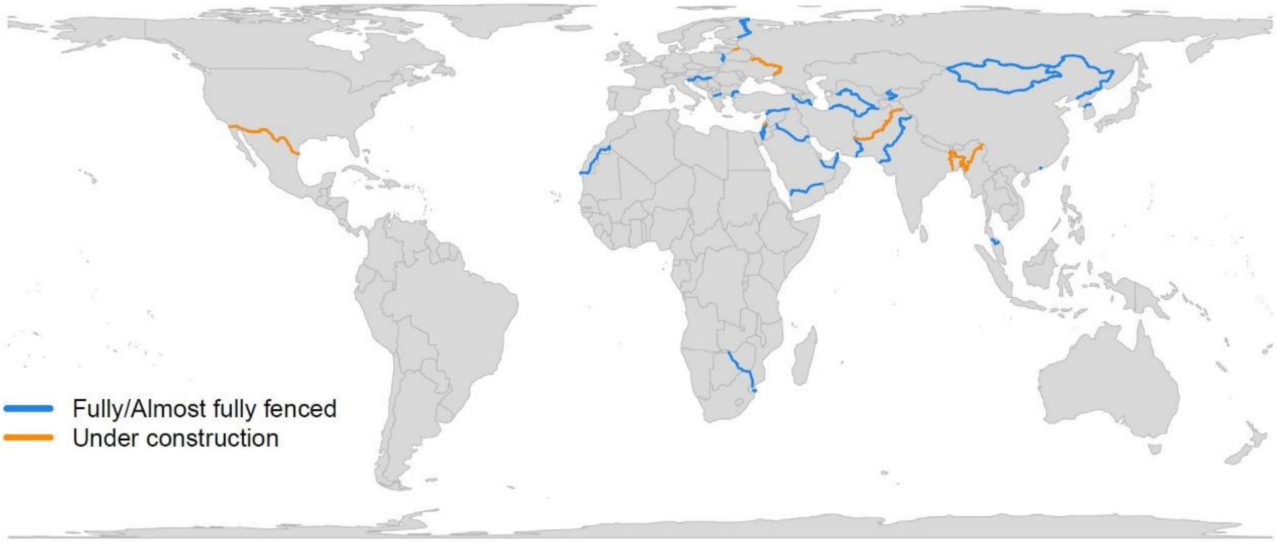


**Figure S7:** Global map of national borders that have physical barriers such as walls and fences across their entire length (blue) and borders where physical barriers are under construction (orange).

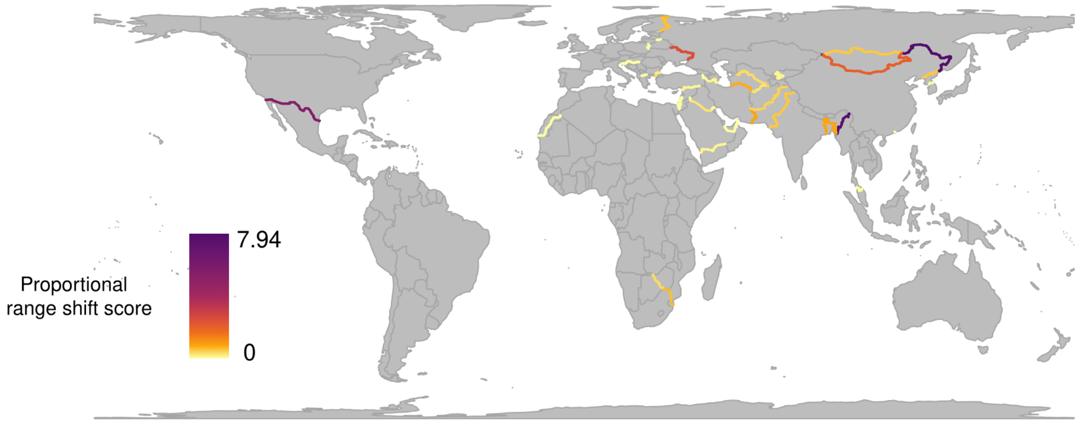


**Figure S8:** Projected range shifts of non-flying mammals (2070, RCP8.5) across borders that are fortified with walls or fences (including those under construction). The proportional range shift score sums together, for all species that cross that border, the proportion of their range that crosses it. As with the number of species projected to cross each border (Fig 4c), the USA-Mexico border, the India-Myanmar border and the China-Russia border rank highest.

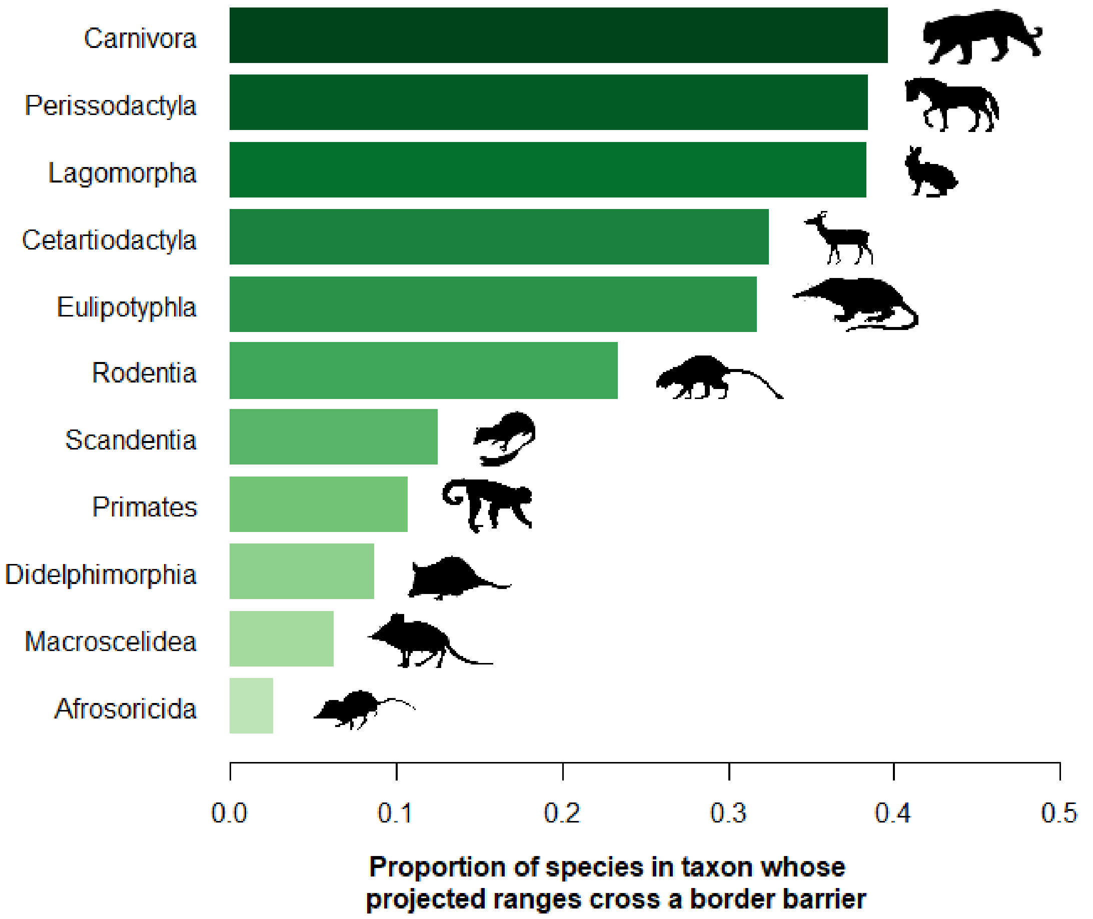


**Figure S9:** Proportion of species in mammalian orders whose ranges are projected to cross a border barrier under climate change (projecting to 2070 under RCP 8.5). The order Chiroptera (bats) was excluded, as well as orders that contained fewer than 10 modelled species.

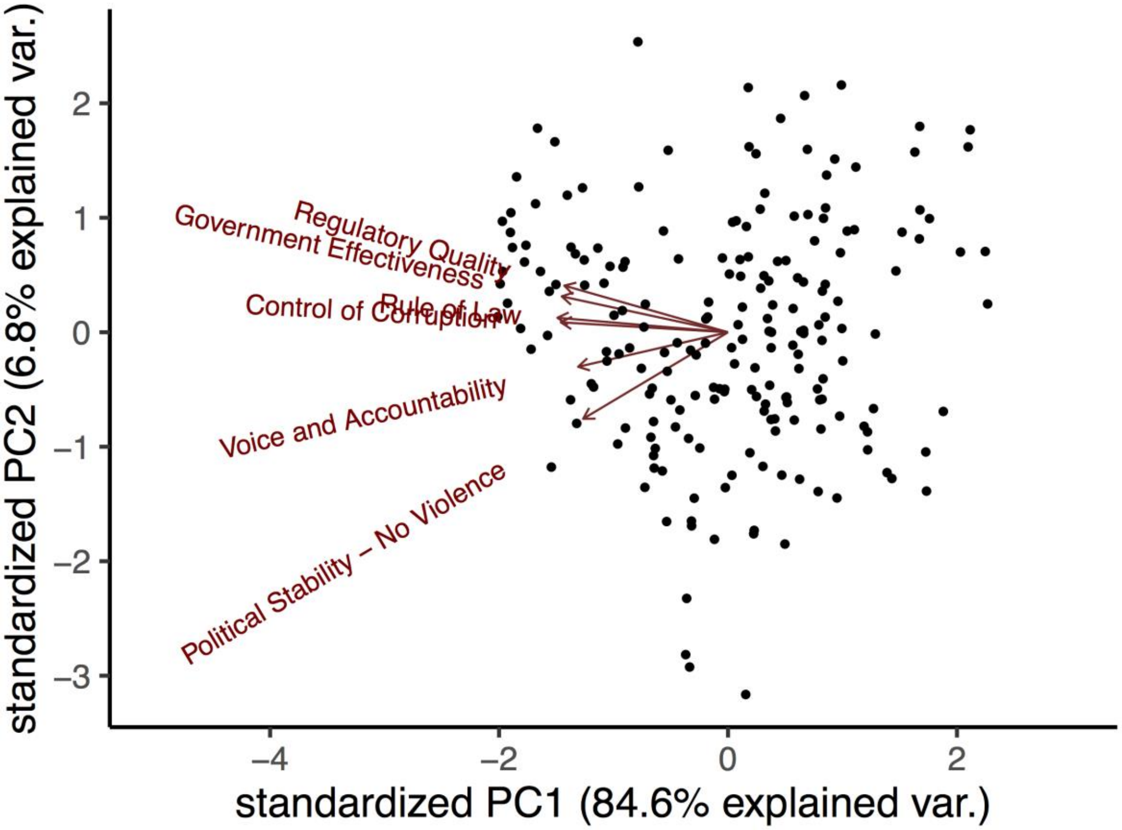


**Figure S10.** Principal Components Analysis (PCA) of the six governance scores provided by the World Bank (15) showing a high level of inter-correlation, supporting our decision to follow (7) and aggregate them into a single indicator of governance quality by taking the mean of all six for each country. Each data point represents a country.

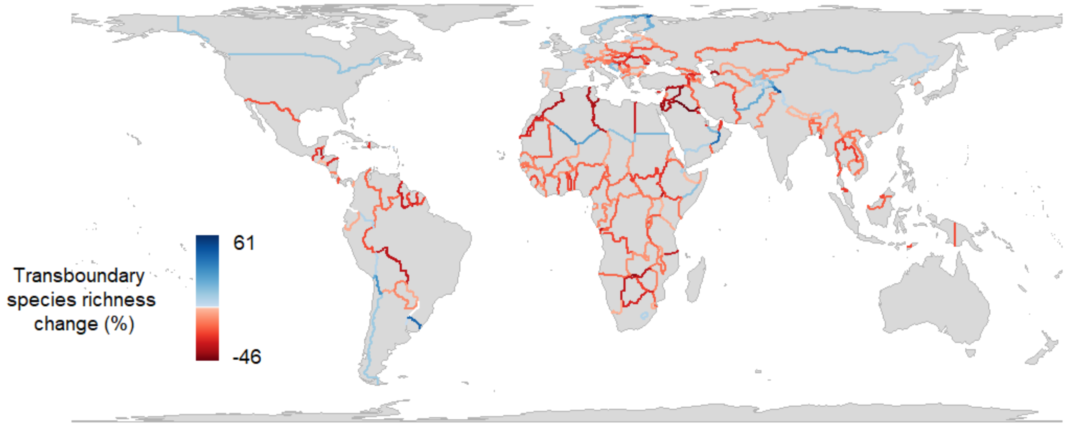


**Figure S11:** Projected percentage change in transboundary species richness (the number of species whose ranges intersect each political border), comparing present day to 2070 under RCP 8.5.

**Table S1.** GLM model coefficient estimates and significance values. In each case the response variable is national level percentage change in species richness by 2070 (averaged across all half-degree grid cells within a country). P values and t values shown are for the slope estimate. · P < 0.1, * P<0.5, ** P < 0.01, *** P < 0.001.

| Taxon | Predictor variable | Scenario | Intercept | Slope | t value | P value |
|---|---|---|---|---|---|---|
| Both | Governance score | RCP 2.6 | -1.876 | 1.330 * | 2.056 | **0.0413** |
| | | RCP 4.5 | -5.268 | 2.642 ** | 3.332 | **0.00105** |
| | | RCP 6.0 | -5.256 | 3.082 *** | 3.701 | **0.000288** |
| | | RCP 8.5 | -9.827 | 3.904 *** | 3.904 | **0.000135** |
| Both | $Log_{10}$ GDP per capita | RCP 2.6 | -7.664 | 1.435 | 1.464 | 0.1452 |
| | | RCP 4.5 | -16.137 | 2.813 * | 2.374 | **0.018819** |
| | | RCP 6.0 | -19.472 | 3.660 ** | 2.942 | **0.00375** |
| | | RCP 8.5 | -27.633 | 4.582 ** | 2.790 | **0.00592** |
| Both | $Log_{10}$ $CO_2$ emissions per capita | RCP 2.6 | -2.411 | 0.742 | 0.846 | 0.398751 |
| | | RCP 4.5 | -5.800 | 1.498 | 1.341 | 0.182 |
| | | RCP 6.0 | -6.213 | 2.212 · | 1.881 | **0.0617** |
| | | RCP 8.5 | -10.844 | 2.403 | 1.472 | 0.143 |
| Mammals | Governance score | RCP 2.6 | 1.060 | 2.580 *** | 3.386 | **0.000886** |
| | | RCP 4.5 | -1.041 | 4.227 *** | 4.656 | **$6.56 \times 10^{-6}$** |
| | | RCP 6.0 | -1.210 | 4.704 *** | 4.799 | **$3.54 \times 10^{-6}$** |
| | | RCP 8.5 | -4.697 | 6.724 *** | 5.324 | **$3.26 \times 10^{-7}$** |
| Mammals | $Log_{10}$ GDP per capita | RCP 2.6 | -14.011 | 3.881 *** | 3.304 | **0.00118** |
| | | RCP 4.5 | -23.424 | 5.806 *** | 4.189 | **$4.64 \times 10^{-5}$** |
| | | RCP 6.0 | -28.136 | 7.009 *** | 4.750 | **$4.54 \times 10^{-6}$** |
| | | RCP 8.5 | -40.086 | 9.246 *** | 4.938 | **$1.99 \times 10^{-6}$** |
| Mammals | $Log_{10}$ $CO_2$ emissions per capita | RCP 2.6 | -0.301 | 2.416 * | 2.308 | **0.0222** |
| | | RCP 4.5 | -2.700 | 3.421 ** | 2.661 | **0.00855** |
| | | RCP 6.0 | -3.297 | 4.474 ** | 3.258 | **0.00136** |
| | | RCP 8.5 | -7.165 | 5.518 ** | 3.036 | **0.00279** |
| Birds | Governance score | RCP 2.6 | -2.771 | 0.950 | 1.419 | 0.158 |
| | | RCP 4.5 | -6.393 | 2.388 ** | 2.887 | **0.00441** |
| | | RCP 6.0 | -6.475 | 2.660 ** | 3.065 | **0.00254** |
| | | RCP 8.5 | -11.448 | 3.860 ** | 3.241 | **0.00144** |
| Birds | $Log_{10}$ GDP per capita | RCP 2.6 | -5.363 | 0.543 | 0.537 | 0.592 |
| | | RCP 4.5 | -13.289 | 1.660 | 1.367 | 0.17343 |
| | | RCP 6.0 | -16.333 | 2.429 · | 1.918 | **0.056978** |
| | | RCP 8.5 | -23.101 | 2.846 · | 1.702 | **0.090801** |
| Birds | $Log_{10}$ $CO_2$ emissions per capita | RCP 2.6 | -3.175 | 0.070 | 0.077 | 0.939 |
| | | RCP 4.5 | -6.922 | 0.664 | 0.577 | 0.565 |
| | | RCP 6.0 | -7.297 | 1.305 | 1.084 | 0.280 |
| | | RCP 8.5 | -12.277 | 1.132 | 0.679 | 0.498 |
